# Proband Whole-Exome Sequencing Identified Genes Responsible for Autosomal Recessive Non-Syndromic Hearing Loss in 33 Chinese Nuclear Families

**DOI:** 10.3389/fgene.2019.00639

**Published:** 2019-07-17

**Authors:** Shushan Sang, Jie Ling, Xuezhong Liu, Lingyun Mei, Xinzhang Cai, Taoxi Li, Wu Li, Meng Li, Jie Wen, Xianlin Liu, Jing Liu, Yalan Liu, Hongsheng Chen, Chufeng He, Yong Feng

**Affiliations:** ^1^Department of Otolaryngology, Xiangya Hospital, Central South University, Changsha, China; ^2^Key Laboratory of Otolaryngology Major Diseases Research of Hunan Province, Changsha, China; ^3^Institute of Molecular Precision Medicine, Xiangya Hospital, Central South University, Changsha, China; ^4^Hunan Key Laboratory of Molecular Precision Medicine, Changsha, China; ^5^Department of Otolaryngology, University of Miami Miller School of Medicine, Miami, FL, United States; ^6^Dr. John T. Macdonald Foundation Department of Human Genetics, University of Miami Miller School of Medicine, Miami, FL, United States; ^7^Hunan Jiahui Genetics Hospital, Changsha, China; ^8^National Clinical Research Center for Geriatric Disorders, Xiangya Hospital, Central South University, Changsha, China

**Keywords:** proband-WES, ARNSHL, nuclear families, similar phenotype, molecular diagnosis

## Abstract

Autosomal recessive non-syndromic hearing loss (ARNSHL) is a highly heterogeneous disease involving more than 70 pathogenic genes. However, most ARNSHL families have small-sized pedigrees with limited genetic information, rendering challenges for the molecular diagnosis of these patients. Therefore, we attempted to establish a strategy for identifying deleterious variants associated with ARNSHL by applying proband whole-exome sequencing (proband-WES). Aside from desiring to improve molecular diagnostic rates, we also aimed to search for novel deafness genes shared by patients with similar phenotype, making up for the deficiency of small ARNSHL families. In this study, 48.5% (16/33) families were detected the pathogenic variants in eight known deafness genes, including 10 novel variants identified in *TMPRSS3* (MIM 605551), *MYO15A* (MIM 602666), *TMC1* (MIM 606706), *ADGRV1* (MIM 602851), and *PTPRQ* (MIM 603317). Apart from six novel variants with a truncating effect (nonsense, deletion, insertion, and splice-site), four novel missense variants were not found in 200 unrelated control population by using Sanger sequencing. It is important to note that none of novel genes were shared across different pedigrees, indicating that a larger sample size might be needed. Proband-WES is a cost-effective and precise way of identifying causative variants in nuclear families with ARNSHL. This economical strategy may be appropriated as a clinical application to provide molecular diagnostics, genetic counseling, and individualized health maintenance measures for patients with ARNSHL at hearing clinics.

## Background

Hearing loss (HL) is one of the most common sensory disorders, affecting nearly 328 million adults and 32 million children worldwide (WHO, 2017). It is estimated that approximately 30,000 Chinese infants are born with congenital non-syndromic HL (NSHL) annually (Ouyang et al., 2009). For congenital or early-onset HL, genetic factor is considered to be the dominant etiology (Nance, 2003; Wang et al., 2018), especially for those with a family history. Among hereditary cases, approximately 70% are NSHL, for which hearing impairment is the exclusive phenotype (Niu et al., 2017; Deng et al., 2018). It is reported that almost 80% of NSHL cases involve autosomal-recessive inheritance with high genetic heterogeneity (Angeli et al., 2012). The major symptom of autosomal-recessive NSHL (ARNSHL) is bilaterally symmetric, severe-to-profound, and prelingual sensorineural HL, which is known to be caused mainly by monogenic mutations (Wu et al., 2016; Deng et al., 2018).

Most families with ARNSHL have small-sized pedigrees with an affected sib pair, affected parent–child pair, or a single affected child (Bademci et al., 2016). Despite the fact that linkage analysis has succeeded in mapping an abundance of genetic diseases over past few decades (Vardarajan et al., 2018), compared to studies associated with large-sized pedigrees, nuclear families with limited generations provide less information for genetic mapping and yield lower statistical power to discern quantitative trait loci (QTL) by linkage analysis (Dyer et al., 2001). Large unsolved families with ARNSHL urgently require a cost-effective method to detect the genetic etiology. The emergence of whole-exome sequencing (WES) offers an unprecedented opportunity to explore causative mutations in patients suffering from extremely heterogeneous genetic disorders (Li et al., 2018), such as ARNSHL.

In present study, we recruited affected sib pair pedigrees with similar phenotypes and performed WES only on probands of each family (so-called proband-WES) to search for candidate deafness-causing variants. Subsequently, we applied Sanger sequencing to verify which variants co-segregate with phenotype among the first degree relatives of the probands.

## Materials and Methods

### Subjects

We recruited 33 nuclear families with ARNSHL from the Otolaryngology Department of Xiangya Hospital, Central South University, China. To ensure the pedigrees from a homogeneous population with ARNSHL, we used the following inclusion criteria: 1) the probands had other affected sibling(s); 2) patients’ parents were asymptomatic; 3) all affected individuals suffered from congenital or prelingual deafness with a severity ranging from severe to profound; and 4) HL was the sole symptom of all patients. All experiments were approved by the ethics committee of Xiangya Hospital, Central South University. Written informed consent was provided by all participants or designated guardians of affected children prior to participation in this study.

Detailed clinical evaluation of each affected subject was performed by an otologist and a geneticist, including medical history investigation, somatoscopy, and temporal bone image examination *via* high-resolution computed tomography (HRCT) and magnetic resonance imaging (MRI) when necessary, so as to exclude syndromic and secondary HL. As part of routine examination, audiological evaluations including auditory brainstem response (ABR), multiple auditory steady-state evoked responses (ASSR), and distortion production otoacoustic emissions (DPOAE) were performed on all affected children. Severity of hearing impairment according to ABR data was classified as subtle (16∼25 dB), mild (26∼40 dB), moderate (41∼70 dB), severe (71∼90 dB), or profound (≥90 dB). Clinical and audiological evaluations indicated that all recruited individuals had severe to profound NSHL, without the evidence of syndromic, secondary, or auditory neuropathy. Peripheral blood samples of all probands and their family members (including parents and siblings) were collected and used to extract genomic DNA according to standard procedures for sequencing and molecular analysis.

### WES

Agilent SureSelect Human All Exon v6 (60 Mb) Kit (Agilent Technologies, Santa Clara, CA, USA) was used for WES, and sequencing was conducted on a HiSeq X10 platform (Illumina, San Diego, CA, USA) in an in-solution hybridization capture system to obtain all coding exons and flanking region sequences according to the manufacturer’s standard protocol.

One microgram of genomic DNA from proband was fragmented into 180–200 bp libraries which were modified, ligated to adaptors, and purified for subsequent PCR amplification. Amplified products were then hybridized to a custom array using the SureSelect^XT^ Target Enrichment System (G7530-90000, Agilent). The hybridized DNA was eluted using magnetic beads, and the final enriched DNA fragments were then re-amplified by bridge-PCR and sequenced simultaneously on an Illumina HiSeq X10 System with 100-bp paired-end sequencing.

The raw sequencing data was filtered to remove low-quality reads [the ratio of bases with low quality scores (less than 19) > 50% and/or ambiguous nucleotide rate >5% and/or adapter contamination >5 bp] and then turned into clean reads. These clean reads were then aligned to the reference human genome sequence (hg19) using the Burrows-Wheeler Aligner (BWA) (Li and Durbin, 2010). The GATK Software (https://www.broadinstitute.org/gatk/) was used for base quality re-calibration and variants calling (McKenna et al., 2010). We also used ANNOVAR software to annotate each variant, including gene name, genomic region, and function.

### Bioinformatics Analysis

**Figure 1:**
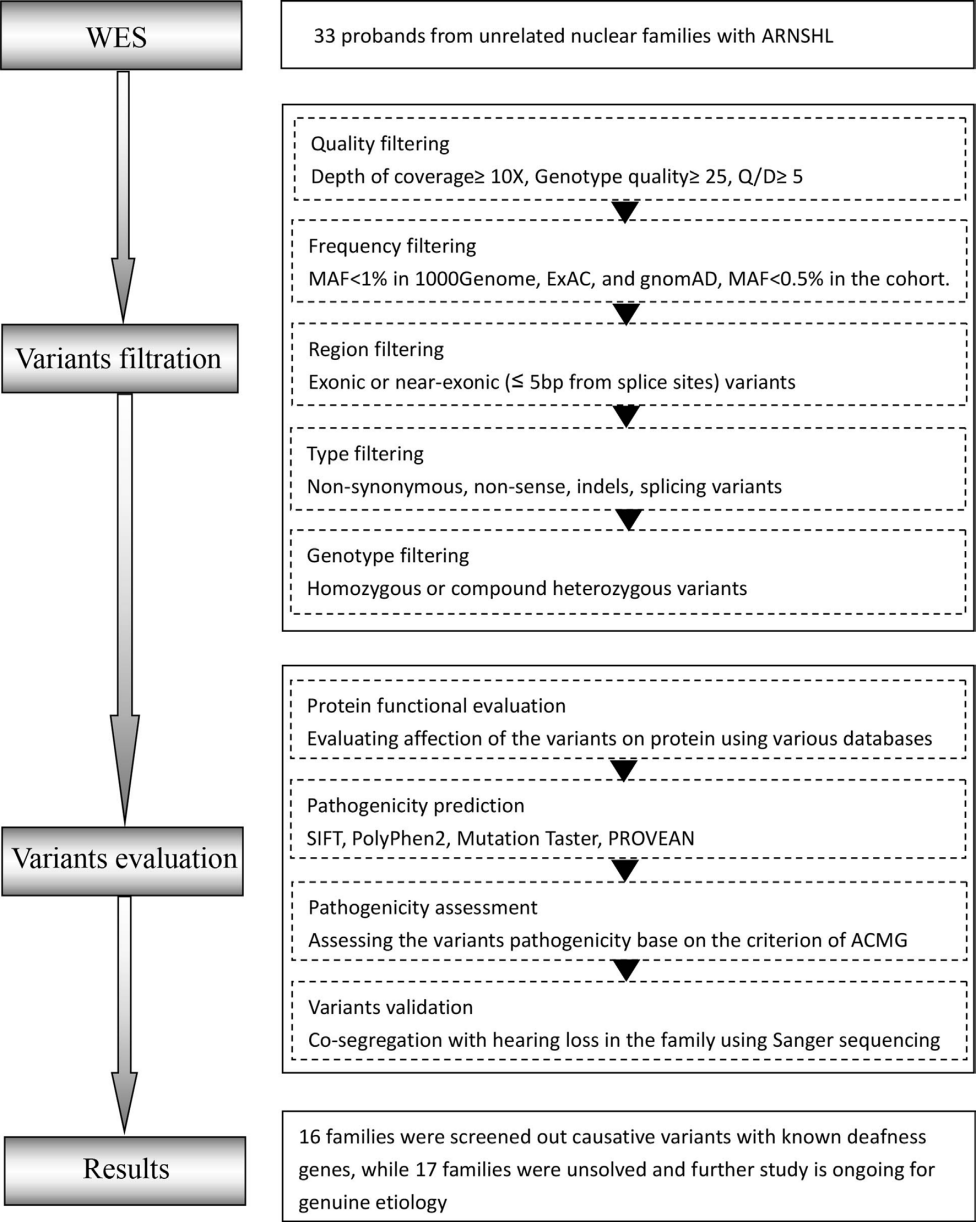
Overall workflow of the proband whole-exome sequencing (proband-WES) pipeline. O/D, Phred-like quality score divided by depth; MAF, minor allele frequency.

The workflow of this study is shown in **Figure 1**.

#### Variants Filtration

The analysis began with quality control (QC) including the coverage and average read depth of all coding exons and intron-exon adjacent regions, genotype quality scores, and QualByDepth (Q/D) scores. The low-quality variants (read depth <10-fold, quality score <25, Q/D score <5) were excluded from downstream analysis. The remaining variants were filtered according to minor allele frequency (MAF) <1% in multiple databases including the Exome Aggregation Consortium (ExAC) (http://exac.broadinstitute.org/), the 1000 Genome Project (http://browser.1000genomes.org), and gnomAD (http://gnomad.broadinstitute.org/). Variants with an allele frequency > 0.5% in the Annoroad Typical Chinese Genomes (ATCG database) were also filtered out. In addition, we produced a deafness-associated gene list (DAGL) by searching the Deafness Variation Database (DVD; http://deafnessvariationdatabase.org/), OMIM database (http://www.omim.org/), and HGMD database (http://www.hgmd.cf.ac.uk/ac/) with keywords “hearing loss” or “deafness” (**Supplementary Table 1**). The filtered variants were further annotated according to whether they appeared in the DAGL or not. Among the filtered variants, all homozygous and putatively compound heterozygous mutations were selected as candidate pathogenic variants.

#### Variant Evaluation

The candidate variants were annotated on the basis of their pathogenicity information in the ClinVar (http://www.ncbi.nlm.nih.gov/clinvar), the DVD and HGMD database. Pathogenic prediction scores were calculated for non-synonymous variants to assess the influence of amino acid substitution on protein structure and function with Polyphen2 (http://genetics.bwh.harvard.edu/pph2), SIFT (http://sift.jcvi.org), MutationTaster (http://mutationassessor.org), and MutationAssessor (http://mutationassessor.org). Only when pathogenic prediction scores were generated by two or more tools, were the non-synonymous variants considered pathogenic. We reclassified variants based on the American College of Medical Genetics and Genomics (ACMG) guidelines into five categories: pathogenic (P), likely pathogenic (LP), uncertain significance (US), likely benign (LB), and benign (B) (Richards et al., 2015).

Homozygous and putatively compound heterozygous variants that were detected in the DAHL or predicted to be “P” by the ACMG guidelines were given priority to further analysis. In unsolved families, homozygous and putatively compound heterozygous variants predicted to be “LP” or “US” by the ACMG guidelines were chosen for further verification.

### Sanger Sequencing

Sanger sequencing was used to confirm candidate variants to determine whether they co-segregated with HL in family members. Primers for the exons of all candidate variants were designed using Primer3 online software (http://primer3.ut.ee/) based on the human genome reference sequence and manufactured by Bioshon Corporation, Changsha, China. The genomic DNA of candidate variants was amplified with primer pairs and then purified by agarose gel electrophoresis following the manufacturer’s protocol. The purified products were sequenced on an ABI 3100 DNA Analyzer (Thermo Fisher Scientific, Waltham, MA, USA). Sequencing data was analyzed using Lasergene version 7.1 (DNASTAR, Madison, WI, USA).

After sequencing, only those variants co-segregated with disease status in all family members were deemed responsible for HL. Finally, we also tested the novel missense variants identified in this study by Sanger sequencing and excluded those which have been detected in 200 unrelated control subjects.

### Structure Modeling

To evaluate the effect of the novel missense variants detected in *TMPRSS3* and *MYO15A* on the structure and function of the proteins, three-dimensional (3D) models of the wild-type and mutant protein structures were built. Since there is no homology model for the protein structures of *TMPRSS3* and *MYO15A* in the Protein Data Bank (PDB), the 3D molecular structures were constructed by the I-TASSER server creating five models per protein structure (Roy et al., 2010; Roy et al., 2012) and the optimal models were visualized using PyMOL software.

## Results

In this study, average coverage of the targeted regions (exons and adjacent 20 bps introns from splice sites) were 99%, 97%, and 94% for the 10X, 30X, and 50X reads, respectively (**Supplementary Table 2**).

According to the filtering criteria mentioned above, 48.5% (16/33) families were found to carry homozygous or compound heterozygous variants in the DAGL. All detected variants were predicted to be P or LP by the ACMG guidelines and met the phenotype–genotype co-segregation. A total of 18 different variants were detected in eight known deafness genes. The known deafness genes with variants detected in this study were *GJB2* (MIM 121011; 21.2%), *TMPRSS3* (MIM 605551; 6.1%), *MYO15A* (MIM 602666; 6.1%), *TMC1* (MIM 606706; 3%), *ADGRV1* (MIM 602851; 3%), *PTPRQ* (MIM 603317; 3%), *SLC26A4* (MIM 605646; 3%), and *OTOF* (MIM 603681; 3%) (**Table 1**). Among them, 55.6% (10/18) were novel variants that apart from six novel variants with a truncating effect (nonsense, deletion, insertion, and splice-site), the other four were missense variants detected in *TMPRSS3 and MYO15A*, respectively, which were undetected in 200 unrelated control subjects.

**Table 1:** The detailed information of pedigrees with pathogenic variants identified in this study.

Family ID	Gene name	mRNA	Variation	Aa Change	Zygosity	Alle Frq. gnomAD	SIFT	Polyphen2	Mutation Taster	ACMG	Reference
HL533	TMPRSS3	NM_024022.2	c.551T > C	p.L184S	Hom.	1.62E-05	0.003	0.99	1	LP	Novel#
M21968	TMPRSS3	NM_024022.2	c.432delA	p.Q144fs	Hom.	3.61E-05	.	.	.	P	Novel
M21958	MYO15A	NM_016239.3	c.3833A > C	p.Q1278P	Comp.Het.	NA	0.053	0.939	0.967	LP	Novel#
	MYO15A	NM_016239.3	c.9876G > T	p.W3292C		1.44E-05	0.003	1	1	LP	Novel#
HL1822	MYO15A	NM_016239.3	c.4351G > A	p.D1451N	Comp.Het.	1.22E-05	0.006	1	1	LP	PMID: 17546645
	MYO15A	NM_016239.3	c.6461G > A	p.C2154Y		NA	0	1	1	LP	Novel#
D008	TMC1	NM_138691.2	c.150delT	p.N50fs	Comp.Het.	NA	.	.	.	P	Novel
	TMC1	NM_138691.2	c.1224+2T > C	NA		NA	.	.	1	P	Novel
HL1121	ADGRV1	NM_032119.3	c.3232G > T	p.E1078X	Comp.Het.	NA	.	.	1	P	Novel
	ADGRV1	NM_032119.3	c.2057_2063del	p.A686fs		NA	.	.	.	P	Novel
HL1222	PTPRQ	NM_001145026.1	c.552delC	p.D184fs	Hom.	NA	.	.	.	P	Novel
M19411	OTOF	NM_194248.2	c.5713-2A > G	NA	Comp.Het.	1.63E-05	.	.	1	LP	PMID: 22575033
	OTOF	NM_194248.2	c.5197G > A	p.E1733K		4.06E-06	0.002	1	1	P	PMID: 19250381
D033	GJB2	NM_004004.5	c.235delC	p.L79fs	Hom.	0.000451	.	.	.	P	PMID: 27308839
D040	GJB2	NM_004004.5	c.439G > A	p.E147K	Comp.Het.	1.22E-05	0.001	1	1	P	PMID: 14676473
	GJB2	NM_004004.5	c.235delC	p.L79fs		0.000451	.	.	.	P	PMID: 27308839
D048	GJB2	NM_004004.5	c.235delC	p.L79fs	Hom.	0.000451	.	.	.	P	PMID: 27308839
HL051	GJB2	NM_004004.5	c.235delC	p.L79fs	Hom.	0.000451	.	.	.	P	PMID: 27308839
M20989	GJB2	NM_004004.5	c.235delC	p.L79fs	Hom.	0.000451	.	.	.	P	PMID: 27308839
M21006	GJB2	NM_004004.5	c.235delC	p.L79fs	Hom.	0.000451	.	.	.	P	PMID: 27308839
M20960	GJB2	NM_004004.5	c.428G > A	p.R143Q	Comp.Het.	NA	0.001	1	1	P	PMID: 11313763
	GJB2	NM_004004.5	c.235delC	p.L79fs		0.000451	.	.	.	P	PMID: 27308839
HL476	SLC26A4	NM_000441.1	c.1546dupC	p.F515fs	Comp.Het.	1.99E-05	.	.	.	P	PMID: 10874637
	SLC26A4	NM_000441.1	c.919-2A > G	NA		3.65E-04	.	.	.	P	PMID: 10874637

#: were novel missense variants that were not found in 200 unrelated control subjects.

NA, not available; Comp.Het., compound heterozygote.

**Figure 2:**
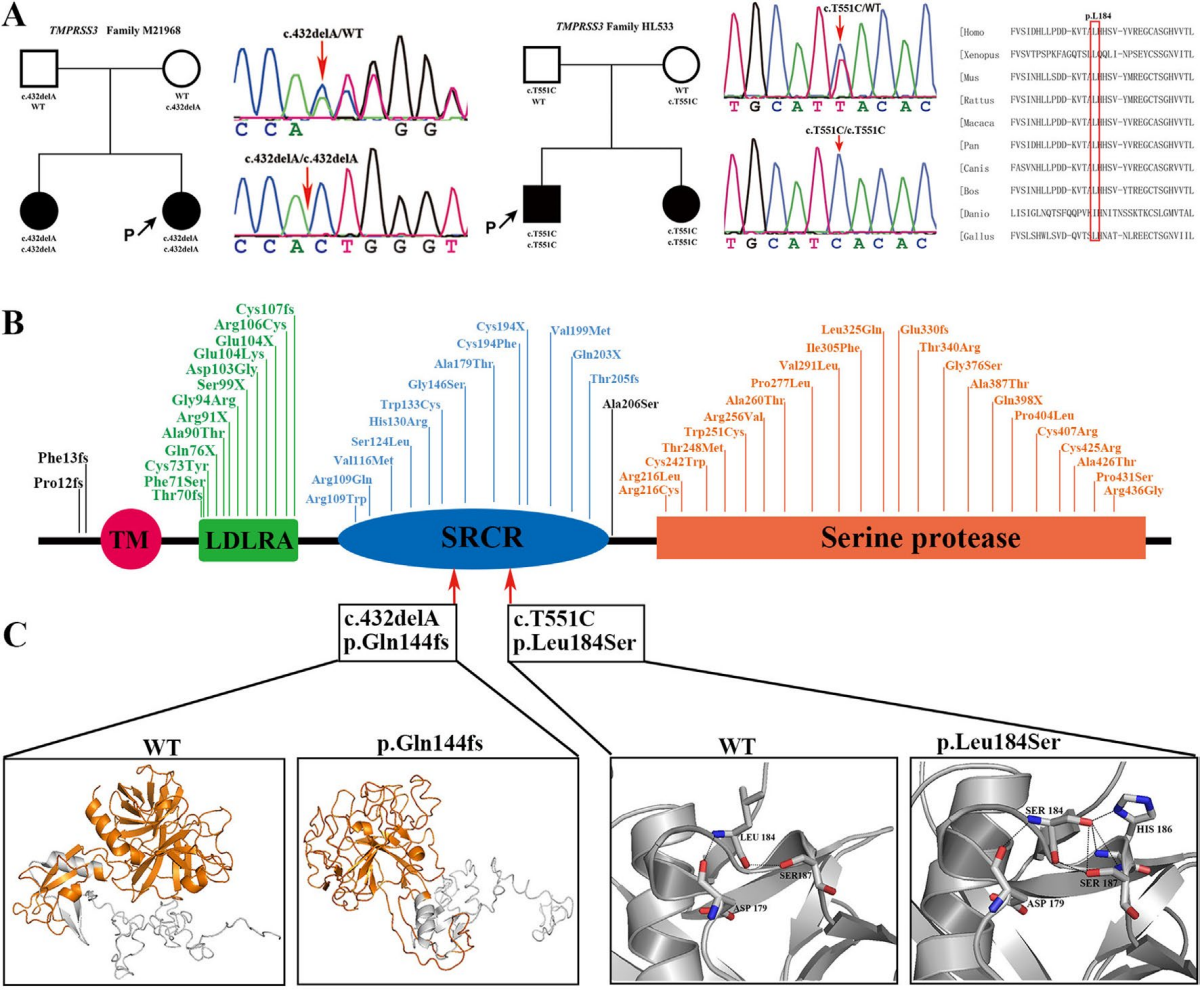
Pedigree information, variation spectrum and three-dimensional protein modeling of TMPRSS3. **(A)** are pedigree charts, DNA sequence chromatograms of the two families with TMPRSS3 mutations as well as the conservative prediction of the c.551T>C (p.Leu184Ser) mutation. **(B)** is the mutant spectrum of TMPRSS3. **(C)** is the 3D models showing that the p.Gln144fs mutation destroys the spatial structure of protein (yellow) and the p.Leu184Ser mutation makes Ser184 form three extra hydrogen bonds with His186 and formed an extra hydrogen bond with Ser187.

A novel homozygous frame-shift mutation and a novel homozygous missense mutation in *TMPRSS3* were identified in two nuclear families, respectively. Both of the affected siblings presented congenital, profound ARNSHL. The *TMPRSS3* c.432delA (p.Q144fs) mutation was predicted to disturb protein spatial structure by destroying the scavenger receptor cysteine-rich (SRCR) domain and the serine protease domain. The *TMPRSS3* c.T551C (p.L184S) mutation resulted in an amino acid substitution at an extremely conservative site in the SRCR domain. The 3D molecular model showed that the *TMPRSS3* c.432delA (p.Q144fs) mutation destroyed spatial structure of the protein by affecting the SRCR and the trypsin-like serine protease domain; for the *TMPRSS3* c.T551C (p.L184S) mutation, the mutant-type serine formed three extra hydrogen bonds with histidine at position 186 and formed an extra hydrogen bond with serine at position 187, which may perturb the protein structure (**Figure 2**). Both of the homozygous mutations [c.432delA (p.Q144fs) and c.T551C (p.L184S)] occurred in the highly conserved SRCR domain of the TMPRSS3 protein, disturbing the function of this transmembrane protein to activate the ENaC sodium channel and interact with extracellular molecules.

**Figure 3:**
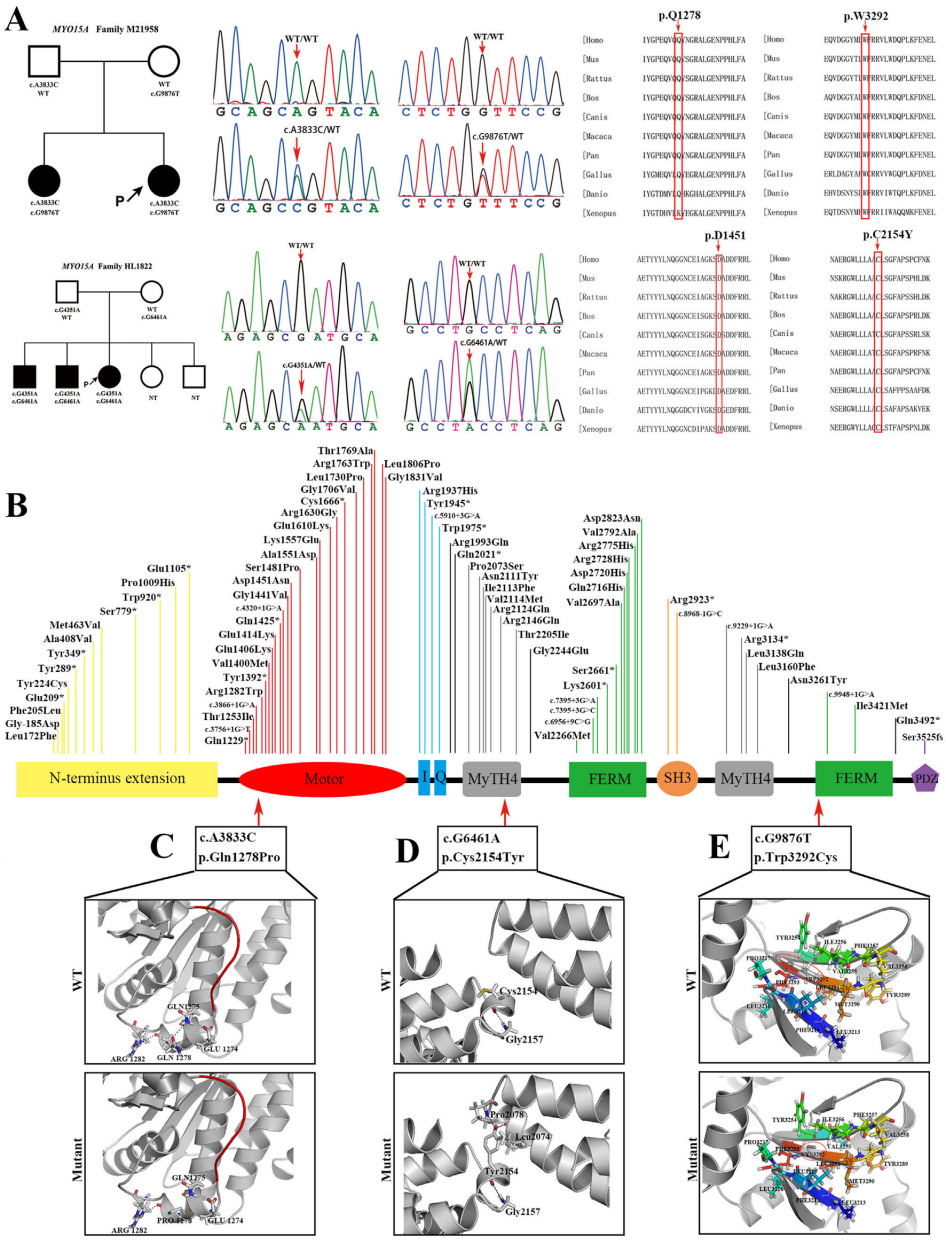
Pedigree information, variation spectrum, and three-dimensional protein modeling of MYO15A. **(A)** are pedigree charts, DNA sequence chromatograms, and the conservative prediction of four MYO15A mutations which is found in two different families. **(B)** is the mutant spectrum of MYO15A. **(C)** is the 3D models showing that the p.Gln1278Pro mutation shortens the side chain and deletes the hydrogen bonds with Gln1275 and Glu1274, which may affect the function of adjacent ATP binding pocket (red); **(D)** the p.Cys2154Tyr mutation lengthens the side chain and adds hydrogen bond with Pro2078 and Leu2074, and **(E)** the p.Trp3292Cys mutation shortens the side chain and destructs the hydrophobic core. NT, not tested.

Two compound heterozygous mutations in *MYO15A* were detected with the following genotypes: c.A3833C (p.Q1278P)/c.G9876T (p.W3292C) occurred in family M21958 and c.G4351A (p.D1451N)/c.G6461A (p.C2154Y) in family HL1822. Multiple sequence alignment (MSA) identified that the four variants in *MYO15A* detected in this study were located at highly conserved sites. Three novel variants, c.A3833C (p.Q1278P), c.G6461A (p.C2154Y), and c.G9876T (p.W3292C), resided in the motor domain, the first MyTH4 domain, and the second FERM domain, respectively (**Figure 3**). Protein modeling revealed that hydrophobic proline was substituted for a hydrophilic amino acid at position 1278 and decreased the number of hydrogen bonds by two compared to the wild-type residue, which may affect the function of adjacent ATP-binding pockets. The motor domain comprises the actin and ATP-binding sites and acts as a molecular dynamic component. The 3D structure modeling of the *MYO15A* motor domain revealed that the c.A3833C (p.Q1278P) mutation weakened the interaction of glutamine 1278 with other amino acids by decreasing the number of hydrogen bonds, which might have a detrimental effect on ATP hydrolysis and actin-binding stability. For the c.G6461A (p.C2154Y) mutation, the cysteine at position 2154 was replaced by tyrosine with a longer side chain, which is predicted to add two extra hydrogen bonds. For the c.G9876T9 (p.W3292C) mutation, tryptophan as a hydrophobic amino acid at position 3292 was surrounded by multiple hydrophobic amino acids that would damage the network of hydrophobic interaction after turning into cysteine (**Figure 3**).

Interestingly, a compound heterozygous mutation in *ADGRV1* with a novel nonsense mutation and a novel frameshift mutation was detected in family HL1121. Their asymptomatic parents carried a heterozygous mutation in *ADGRV1*, respectively. Mutations in *ADGRV1* were related to Usher syndrome 2C. The two affected siblings may be too young to present retinal degeneration and vestibular dysfunction (**Table 1**).

Although several candidate new deafness genes were identified in this study, there were no such genes shared among unrelated pedigrees. Our results indicate that a larger sample size might be needed in further study.

## Discussion

It is challenging to detect causative mutations in ARNSHL due to: 1) small family size with limited generations, 2) high clinical and genetic heterogeneity, and 3) a large portion of non-recurrent variants as a result of large population mobility and low rates of consanguineous marriage. Therefore, the present study aimed to establish a strategy for detecting pathogenic variants causing ARNSHL and identify new genes shared by various pedigrees with similar phenotype, and then provide genetic counseling and health guidance for those individuals with deafness-causing variants identified.

The emergence of next-generation sequencing (NGS) facilitates the discovery of new causative genes and the elucidation of the genetic mechanisms of hereditary diseases. Targeted sequencing panel, WES, and whole-genome sequencing (WGS) are all applications of NGS. However, there is a major limitation of targeted sequencing panel in which only few known genes can be detected and panel requires revalidation after the addition of new genes, making it unsuitable for highly heterogeneous diseases in which new disease-causing genes are constantly being identified (Bademci et al., 2016). It is well known that new deafness-causing genes have been identified every year, which raises an obstacle for the application of panel in clinical deafness etiology detection. Though WGS is the most comprehensive means of detection genetic etiology at present, its high cost makes it unsuitable for population-based studies of Mendelian disease. WES has been widely used as an alternative method to detect causative variants in genetic diseases, especially for Mendelian disorders (Lee et al., 2014). Though less than 2% of the entire human genome contains protein-coding regions, approximately 85% of pathogenic mutations in Mendelian diseases have been identified in these regions (Botstein and Risch, 2003). WES is capable of sequencing nearly all exons in the protein-coding regions of the human genome (Ng et al., 2009) and has innovated etiologic research in the field of human genetic disorders with comprehensive genetic analysis and the identification of novel disorder-causing genes, especially for diseases with extreme heterogeneity. Lee et al. reported that the greater number of disease-causing genes of a Mendelian disease that has been identified, the higher molecular diagnostic rate, could be achieved using WES (Lee et al., 2014). There are over 70 genes with more than 100 loci associated with ARNSHL (http://hereditaryhearingloss.org/); screening these loci would allow to achieve a high molecular diagnostic rate in the deafness cohort. Therefore, patients with ARNSHL are considered to be appropriate for WES because there is no exclusive genetic test available for this highly genetically heterogeneous disorder.

Determining a method by which to determine the pathogenicity of variants is one of the major obstacles for proband-WES. Fortunately, there are limited candidate variants satisfied with autosomal recessive inheritance pattern based on the data of proband-WES, which require further verification in pedigree members. Moreover, plenty of databases gathering all detected variants and several tools predicting the pathogenicity of variants offer powerful resources for genetic diagnosis. Many reference databases, such as the 1000 Genomes Project (1000 Genomes Project Consortium et al., 2015), the exome aggregation consortium (ExAC) (Walsh and Thomson, 2017), and the HapMap Project (Willer et al., 2006), provide vast information for identifying human genetic variations and serve as key components in genetic research by achieving sequence alignment and genotype imputation, evaluating MAF of variants, and filtering potential neutral variants. Multiracial samples and data from these databases allow for broad sharing and provide a wide representation of human genetic variation, which allows for the identification of connections between variants and hereditary diseases, making it easy to filter variants and make up for the drawbacks of nuclear families for linkage analyses. Furthermore, the ACMG developed a detailed set of guidelines for the interpretation of the functional significance of sequence variants generated by next-generation sequencing (Richards et al., 2015), facilitating the precise interpretation of variants pathogenicity.

WES for nuclear families can be conducted as trio-WES (both the proband and his/her related parents sequenced simultaneously) to identify *de novo* variants or as proband-WES (only the proband sequenced). *De novo* variants occurring in the proband are frequently heterozygous and likely result in autosomal dominant inheritance. In fact, two non-twin siblings have little chance of generating the same *de novo* variant causing deafness. A large-scale clinical study showed that, except for *de novo* variants, trio-WES did not yield a higher molecular diagnostic rate than proband-WES (Lee et al., 2014). Therefore, we performed proband-WES on the nuclear families with ARNSHL to detect potentially causative variants combined with parents-child Sanger sequencing (both parents and their siblings were sequenced simultaneously) to verify the candidate variants.

In the present study, the strategy of proband-WES yielded a high molecular diagnostic rate (16 of 33 cases, 48.5%), indicating that our methods succeeded in identifying the genetic etiology for a large portion of ARNSHL families. It is worth noting that some putatively compound heterozygous variants were proven by Sanger sequencing to be transmitted from one of the parents (data not shown), suggesting that these pseudo-compound heterozygous variants were benign and therefore should be removed from consideration as pathogenic in the affected subjects. Therefore, any compound heterozygous variants tested in the proband must be validated as having been transmitted from both parents.

In family HL1121, we detected a novel c.G3232T (p.E1078X) nonsense variant and a novel c.2057_2063del (p.A686fs) frameshift variant in *ADGRV1*, which segregated with hearing loss in this family. *ADGRV1*, a gene that is crucial for the development of hair cells, can result in Usher syndrome 2C, which is characterized by congenital moderate-to-severe hearing loss, retinal degeneration in the second decade of life or later, and normal vestibular function (Yan et al., 2018). This intriguing finding may be associated with the fact that these affected members might be too young to present retinal degeneration, which generally begins to appear in patients with Usher syndrome during puberty. Our results allow for the recommendation to acquire Braille alphabet competency in advance of the onset of blindness, which would improve social-life adaptability and quality of life after becoming speech- and hearing-impaired, as well as blindness.

In the current study, three known deafness genes (*GJB2, TMPRSS3, and MYO15A*) were detected in several pedigrees, suggesting that the strategy of proband-WES can be successfully applied to search for novel ARNSHL genes shared by multiple families with similar phenotype. However, no new gene was found to be shared by multiple families, which may be due to the limited sample size. As a result, we plan to expand the sample size in future. Additionally, there are still some pedigrees that have not identified a genetic etiology, which may be due to: 1) effect of environmental factors or combined effect of environmental and genetic factors and 2) variants located in intron areas or other types of variants that are beyond the detectability of WES.

According to the results of the present study, the strategy of proband-WES is a cost-effective and highly efficient testing procedure for ARNSHL families. Thus, this strategy can be applied to numerous ARNSHL patients at hearing clinics to yield a genetic diagnosis. Acquiring a genetic diagnosis of HL may offer a wealth of benefits such as making individualized health instruction, providing genetic counseling, and evaluating the recurrence rate of HL, offering detailed information for pre-natal diagnosis and preimplantation genetic diagnosis (PGD), as we have validated in a previous study (Deng et al., 2018).

## Conclusion

ARNSHL patients with similar phenotypes may carry the same genetic factors, which can be identified by proband-WES with population-based studies. In the present study, 48.5% (16/33) of nuclear families with ARNSHL yielded a molecular diagnosis. Namely, the strategy of proband-WES works well to identify genetic etiology for families with ARNSH, which is the basis of providing genetic counseling and personalized health maintenance measures and preventing the transmission of deafness genes.

## Data Availability

The raw data supporting the conclusions of this manuscript will be made available by the authors, without undue reservation, to any qualified researcher.

## Author Contributions

SS is responsible for the design of this study, acquisition, analysis and interpretation of data for the work, as well as drafting the work; JL revised the draft critically for important intellectual content; CH and YF provided approval for publication of the content; WL, ML, JW, XLL, JL, and YL collect the detailed information and blood samples of pedigrees; HS performs audiometric tests on all patients as well as their family members; XZL, XC, LM and TL agree to be accountable for all aspects of the work in ensuring that questions related to the accuracy or integrity of any part of the work are appropriately investigated and resolved.

## Conflict of Interest Statement

The authors declare that the research was conducted in the absence of any commercial or financial relationships that could be construed as a potential conflict of interest.
